# Affect Across the Wake-Sleep Cycle

**DOI:** 10.1007/s42761-023-00204-2

**Published:** 2023-08-02

**Authors:** Pilleriin Sikka, James J. Gross

**Affiliations:** 1 Department of Psychology, Stanford University, 450 Jane Stanford Way, Stanford, CA 94305 USA; 2 Department of Psychology, University of Turku, 20014 Turku, Finland; 3 Department of Cognitive Neuroscience and Philosophy, University of Skövde, 541 28 Skövde, Sweden

**Keywords:** Affect dynamics, Mind-wandering, Sleep, Dreaming, Affect coherence, Affect regulation

## Abstract

Affective scientists traditionally have focused on periods of active wakefulness when people are responding to external stimuli or engaging in specific tasks. However, we live much of our lives immersed in experiences not related to the current environment or tasks at hand—mind-wandering (or daydreaming) during wakefulness and dreaming during sleep. Despite being disconnected from the immediate environment, our brains still generate affect during such periods. Yet, research on stimulus-independent affect has remained largely separate from affective science. Here, we suggest that one key future direction for affective science will be to expand our field of view by integrating the wealth of findings from research on mind-wandering, sleep, and dreaming to provide a more comprehensive account of affect across the wake-sleep cycle. In developing our argument, we address two key issues: affect variation across the wake-sleep cycle, and the benefits of expanding the study of affect across the full wake-sleep cycle. In considering these issues, we highlight the methodological and clinical implications for affective science.

Affect colors our experiences not only when we are awake but also when we are asleep. Yet, to date, affective science has focused nearly exclusively on active wakefulness and has largely overlooked affect during other states—from mind-wandering during (resting) wakefulness to dreaming during sleep. This is illustrated by the fact that fewer than 10% of the articles in the first three years of *Affective Science* have focused on sleep and dreaming (all published in the special issue on sleep in June 2022), and none have addressed mind-wandering.

Here, we suggest that one key future direction for affective science will be to expand our field of view by integrating research on mind-wandering, sleep, and dreaming in order to provide a more comprehensive account of affect across the wake-sleep cycle. We advance our argument by addressing two key issues: (1) affect variation across the wake-sleep cycle, and (2) the benefits of expanding the study of affect across the wake-sleep cycle. In considering these issues, we highlight the methodological and substantive implications of findings for affective science.

## Affect Variation Across the Wake-Sleep Cycle

We use the term *affect* as an umbrella term covering various valenced states (e.g., emotions and moods) and encompassing multiple components, such as subjective experience, behavior/expression, and physiological responses. Like many other embodied processes, affect is regulated by the approximately 24-h circadian cycle as well as by environmental factors (e.g., activities we are engaged in, such as eating, exercising, socializing) (e.g., Stone et al., 2006). This means that affect varies not only across, but also within, wakefulness and sleep. Considering this variation is important because it enables an understanding of the mechanisms underlying normal and abnormal variation in affect.

### Affect During Wakefulness

Affective science traditionally has focused on relatively alert waking moments. However, affect during wakefulness varies depending on the time of the day or night. For example, positive affect is typically highest during the daytime (Clark et al., 1989; Murray et al., 2002; Stone et al., 2006), while negative affect is highest during the nighttime (Emens et al., 2020; Miller et al., 2015), although the times of affect peaks and troughs differ from study to study. It has been suggested that variation in the endogenous circadian rhythms of affect may underlie affective disorders (Emens et al., 2020; Perlis et al., 2016). Thus, it is crucial to measure affect across a broader time window than is usually done (e.g., beyond 9 pm) covering both active and resting wakefulness.

Also, affect typically has been studied in relation to external stimuli or specific tasks. Yet, we spend almost half of our waking hours immersed in thoughts and experiences not related to the current environment or task at hand (Kane et al., 2017; Killingsworth & Gilbert, 2010). Such stimulus-independent or task-unrelated experiences are typically referred to as mind-wandering or daydreaming (Seli et al., 2018; Smallwood & Schooler, 2015). Affect is central to these experiences, both during active wakefulness, when our minds wander away from the particular task we are supposed to be focused on (e.g., thinking about the argument we had with our partner when listening to a presentation), and during resting wakefulness (e.g., daydreaming about the upcoming holiday when relaxing after the working day).

Research shows that affect varies depending on whether we are focused on the here and now or our minds are wandering. Studies indicate that people experience more positive affect when they are focused on what they are doing at a particular moment, rather than when they are thinking about something else (Killingsworth & Gilbert, 2010; Mills et al., 2021), although contrary evidence also exists (e.g., Gross et al., 2021). Even though mind-wandering is often linked to negative affect, how positive or negative people feel depends on the content of mind wandering. For example, whereas rumination and worry are associated with negative affect (DuPre & Spreng, 2018), when people’s minds wander to thoughts that are more interesting (e.g., Franklin et al., 2013) and related to the future (e.g., rather than the past) (Ruby et al., 2013) they experience more positive affect*.* Mind-wandering also varies between individuals: whereas in non-clinical populations mind-wandering is typically characterized by a mild positivity bias (Fox et al., 2018; Smallwood & Andrews-Hanna, 2013), in people suffering from affective disorders mind-wandering is more negatively tinged (DuPre & Spreng, 2018; Hoffmann et al., 2016).

### Affect During Sleep

Sleep is a neurophysiological and behavioral state characterized by perceptual disengagement from and unresponsiveness to the external environment (Carskadon & Dement, 2017). Like wakefulness, sleep is a heterogeneous state, consisting of non-rapid eye movement (NREM) and rapid eye movement (REM) sleep, each having its own neurophysiological, cardiorespiratory, and behavioral characteristics. Although the brain is largely unresponsive to environmental stimuli during sleep (unless the stimuli are strong enough, as in the case of an alarm clock or a crying baby), it remains active and generates affect. Thus, sleep is characterized by stimulus-independent affect, akin to that occurring during mind-wandering in wakefulness (Christoff et al., 2016). It is possible to observe and measure affect-related behavior (especially in individuals with parasomnias, who act out their dreams; Siclari et al., 2020) and physiological responses (e.g., heart and breathing rate, skin conductance) during sleep. Such affect-related behaviors and autonomic activity seem to vary across different sleep stages and time of night (e.g., Trinder et al., 2001) and within the course of a single REM sleep episode (Masset et al., 2022). However, most of our knowledge of affect during sleep derives from the study of subjective affective experiences, i.e., affective dream experiences. These experiences occur during both REM and NREM sleep, but, to date, we lack the evidence as to whether we dream during the entire duration of sleep or only during some periods of it.

As in wakefulness, there is considerable variation in dream affect. Affective dream experiences have been found to vary depending on the time of night and sleep stage. For example, affective dream experiences during the early-morning period (vs. early-night period), and during REM (vs. NREM) sleep are often more negative (Sikka et al., 2018; Smith et al., 2004). Importantly, results differ depending on which method has been used to measure affect. When the affective content of narrative dream reports is analyzed (either by external judges or natural language processing software), only about a third of dreams appear to contain affect and those that do are mostly negatively biased (Domhoff, 2017; Zadra & Domhoff, 2017). When participants rate the extent to which they experienced affect in the preceding dream using affect rating scales, most dreams are affective and affective valence is more balanced or even positive (Schredl & Doll, 1998; Sikka et al., 2014, 2017). These discrepancies are found even when the same dream experiences (in the same participants from the same nights) have been analyzed with these two methods in parallel (Schredl & Doll, 1998; Sikka et al., 2014, 2017, 2021). This indicates that the verbal expression of affect and self-ratings of affect may capture different aspects of the same underlying phenomenon or they may reflect entirely different phenomena (Sikka, 2020), a conclusion also reached in studies on waking affect (Kross et al., 2019; Sun et al., 2020). Specifically, whereas analysis of narrative reports reflects affective language use, self-ratings of affect reflect evaluation of one’s affective experiences, and it is an open question to what extent these two methods capture the subjective affective experience (Kross et al., 2019; Sikka, 2020; Sikka et al., 2017; Sun et al., 2020).

### Affect Across Wakefulness and Sleep

**Fig. 1:**
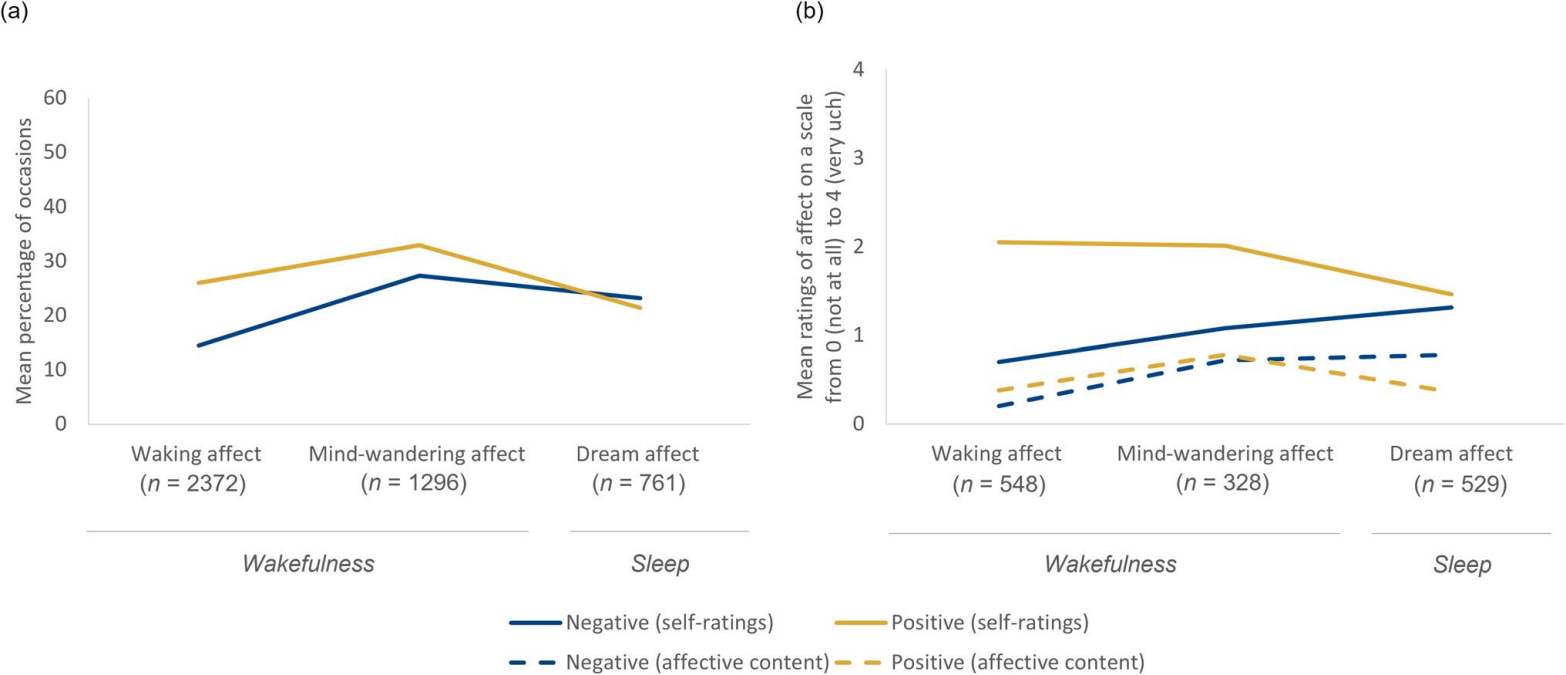
Affect Varies Across the Wake-Sleep Cycle. *Note*. Affect varies across the wake-sleep cycle—from active wakefulness (when we are typically engaged in stimulus-dependent and/or task-related thoughts and experiences) to mind-wandering (characterized by stimulus-independent and/or task-unrelated thoughts and experiences) to dreaming during sleep (during which we are largely disconnected from the current environment). Existing studies suggest that the positivity bias—relative difference in positive vs negative affect—decreases across the wake-sleep cycle. Affect also differs depending on the method used to measure affect, that is, whether participants themselves rate the affect they experience using self-rating scales (referred to as self-ratings) or whether the affective content of narrative reports has been analyzed (referred to as affective content). Figures illustrate the findings from the experience sampling studies of (a) Gross et al. (2021) and (b) Sikka et al. (2021). **a** In Gross et al. (2021), 131 participants were probed three times during the day and two times during the night across 7 days about the affective nature of their experiences at that particular moment. **b** “The dynamics of affect across the wake-sleep cycle: From waking mind-wandering to night-time dreaming” by Sikka, P., Valli, K., Revonsuo, A., & Tuominen, J. (2021). *Consciousness and Cognition, 94*, 103189. 10.1016/j.concog.2021.103189. CC BY 4.0 (https://creativecommons.org/licenses/by/4.0/)

Given the variation of affect during wakefulness and sleep, one question is how similar or different affect is across these states. On the one hand, it has been argued that experiences during wakefulness and sleep lie on a continuum and are phenomenologically similar (Christoff et al., 2016; Fox et al., 2013; Klinger, 1971; Windt, 2021). On the other hand, there seem to be consistent differences in affect across the wake-sleep cycle. Specifically, dream affect is less positive than waking affect (Carr & Nielsen, 2015; Carr et al., 2016; Gross et al., 2021; Sikka et al., 2021) and people experience more fear and anger in dreams than in wakefulness (Nielsen et al., 1991; Sikka et al., 2019). In fact, the positivity bias characteristic to waking experiences (Diener et al., 2015, 2018; Mills et al., 2021) seems to decrease across the wake-sleep cycle—from active wakefulness to mind-wandering to sleep (Gross et al., 2021; Raffaelli et al., 2023; Sikka et al., 2021) (see Fig. 1). This may be due to the gradual decrease of activation of the frontoparietal control network that is involved in cognitive control and affect regulation (Fox et al., 2013). This explanation is supported by research on lucid dreams—dreams in which people are aware that they are dreaming—which are more positive than non-lucid dreams (e.g., Schredl et al., 2022). One explanation for the greater positivity of lucid dreams may be the increased activation of cognitive control areas presumably reflecting enhanced affect regulation processes (Baird et al., 2019; Sikka et al., 2021). Given that a reduced positivity bias (or existence of a negativity bias) is characteristic of affective disorders in wakefulness (e.g., Moore & Fresco, 2012; Williams, 2016), understanding the mechanisms underlying the decreased positivity bias during sleep and dreaming may provide insights into the pathophysiology of various mental disorders.

## Benefits of Expanding the Study of Affect Across Wake-Sleep Cycle

Studying affect across active and resting wakefulness (including mind-wandering), as well as across sleep (including dream affect), will enable a better understanding of underlying affective mechanisms. In this section, we discuss how we can leverage this variation across the wake-sleep cycle to address fundamental questions about affect. We highlight three such questions (of many)—affect dynamics, affect coherence, and differences between affect generation and affect regulation.

### Affect Dynamics

There have been repeated calls to focus research efforts on understanding the temporal dynamics of affect (Davidson, 2015; Kuppens, 2015). However, one constraint is that we miss much of the data if we are sampling at a very low frequency or only during a particular time in the wake-sleep cycle. If we want to know how affect at one point in time is associated with affect at another point in time, we need to sample affect not only across different states within wakefulness but also across sleep. This is especially important given that affect during mind-wandering and dreaming is associated with waking affect. For example, negatively valenced mind-wandering predicts lower mood and symptoms of psychopathology (DuPre & Spreng, 2018; Poerio et al., 2013). Similarly, negative dream affect predicts negatively valenced post-sleep waking affect (Mallett et al., 2022; Schredl & Reinhard, 2009–2010; Sikka et al., 2022). A prominent example is nightmares: negative affect experienced during nightmares often continues into wakefulness and contributes to daytime distress and to various mental health disorders (Levin & Nielsen, 2007, 2009), even to increased risk for suicide (Sandman et al., 2017). Moreover, sleep and dreaming have been argued to play an important role in affect regulation (e.g., Goldstein & Walker, 2014; Levin & Nielsen, 2007, 2009). While there has been a wealth of research on affect regulation in wakefulness (Gross, 2015; Gross, 2023), we know little about how sleep and dreaming help regulate subsequent waking affect and affect regulation. This is unfortunate, because to understand how affect changes or persists over time, the mechanisms underlying these changes, and how these changes relate to ill-being and well-being, we need to study affect and affect regulation across the wake-sleep cycle, and ideally, across several wake-sleep cycles.

### Affect Coherence

An ongoing debate in affective science concerns the degree of coherence among different components of affect (i.e., subjective experience, behavior/expression, physiological responses) (Brown et al., 2019; Petrova et al., 2021; Van Doren et al., 2021). Although these components are assumed to be at least loosely coupled (Gross, 2015), it is unclear whether and to what extent changes in one component necessarily involve changes in other components. Studying affect during sleep provides an opportunity to address these issues. Although REM sleep is characterized by muscle atonia (i.e., functional paralysis of skeletal muscles, except for those controlling eye movement), studies have shown that people do exhibit affective facial expressions during sleep. Yet, these expressions are not necessarily tied to affective dream experiences, at least not in healthy participants (Clé et al., 2019; Maranci et al., 2021; Rivera-García et al., 2019). With regard to physiological responses, whereas some studies have reported associations between dream affect and autonomic activation during REM (e.g., Hauri and Castle, 1973; Paul et al., 2019), evidence for these associations is weak (Nielsen & Zadra, 2011; Schredl, 2018). Thus, existing research suggests that affective dream experiences are not necessarily consistently coupled with specific behavioral or physiological responses. It is possible that this is due to methodological challenges, i.e., the fact that we are necessarily relying on retrospective and aggregated assessment of affective dream experiences, rather than moment-by-moment measurement (Mauss et al., 2005). However, research shows that during lucid dreaming people not only can signal to researchers that they are having a dream (by using a particular pattern of eye movements), but can perform agreed-upon behaviors (e.g., Erlacher et al., 2014) and even answer specific questions asked by researchers (Konkoly et al., 2021). Thus, future research relying on lucid dreaming enabled communication may provide a means for participants to report and rate affective experiences as they are happening during sleep.

### Differences Between Affect Generation and Affect Regulation

Another question in affective science is the extent to which the processes of affect generation and affect regulation can be distinguished (Gross & Barrett, 2011). Given affective science’s historical focus on affect during active wakefulness, it has been difficult to address this question because in wakefulness affect generation typically involves (at least to some extent) affect regulation, especially in a social context, such as in an experimental situation (e.g., Zaki & Williams, 2013). Given the relative deactivation of cognitive control areas during sleep, and lack of control over normal dream content, the study of affect during sleep may provide insights into affect generation relatively uninfluenced by affect regulation processes. Thus, studying affect across different states along the wake-sleep continuum characterized by increasingly reduced activation of cognitive control regions—from active wakefulness to resting wakefulness to sleep—may help clarify the role of affect regulation in shaping affect, although there may also be other factors that account for differences in affect across these states. It also seems possible that studying affect change across the wake-sleep cycle may help us define and measure implicit (as opposed to explicit) affect regulation (Braunstein, et al., 2017) as separate from affect generation. Several theories (Bennion et al., 2015; Goldstein & Walker, 2014) posit that sleep may be a period during which implicit affect regulation processes (akin to fear extinction) occur, and this impacts our waking affect. Testing these ideas may help to clarify the neurophysiological mechanisms of implicit regulation.

## Conclusions

Like waves in the sea, affective experiences ebb and flow across the wake-sleep cycle. Just as measuring the wave only at its crest or trough will give only a partial understanding of wave dynamics, so too measurement of affect only during (active) wakefulness fails to provide a full account of affect dynamics. We have argued that the field of affective science stands to benefit from broadening its field of view and extending the study of affect not only across the full spectrum of wakefulness but also across the full wake-sleep cycle. This will help not only to achieve a more comprehensive understanding of affect but also to address questions that may be difficult, or impossible, to answer by studying affect only during (active) wakefulness.

## Additional Information

### Data Availability

Data sharing not applicable to this article as no datasets were generated or analyzed during the current study.
